# Clinical and Biochemical Parameters in Relation to Serum Fetuin-A Levels in Overweight and Obese with and without Metabolic Syndrome in the North-eastern States of Indian Population

**DOI:** 10.31662/jmaj.2024-0104

**Published:** 2024-09-13

**Authors:** Sruti Eswar, Balaji Rajagopalan, Kenyi Ete, Srinivasa Nageswara Rao Gattem

**Affiliations:** 1 Department of Biochemistry, Sri Balaji Vidyapeeth (Deemed-to be-University), Puducherry, India; 2 Department of Biochemistry, SSSMC&RI, Sri Balaji Vidyapeeth (Deemed-to be-University), Puducherry, India; 3 Department of Biochemistry, Tomo Riba Institute of Health & Medical Sciences, Arunachal Pradesh, India

**Keywords:** Serum Fetuin-A, Overweight, Obesity, Metabolic Syndrome

## Abstract

**Introduction:**

**:** In India the prevalence of metabolic syndrome has dramatically increased. Hepatokines have gained considerable interest, and the role of fetuin-A in overweight and obesity incompletely understood. Therefore, this study aimed to investigate the role of serum fetuin-A in overweight and obese adults with and without metabolic syndrome in the northeastern Indian population.

**Methods:**

This comparative study included 200 subjects (50 control, 50 overweight, 50 obese without metabolic syndrome and 50 obese with metabolic syndrome) aged 20-70 years. Lipid profile and fasting blood glucose, were measured using a fully automated analyzer. ELISA was employed to measure serum fetuin-A levels. Statistical analyses were conducted using SPSS versions 23.0. Furthermore, *t*-test was used to analyze the numerical investigational data of the present study. Chi squared test for the categorical data, and Pearson’s correlation for the correlation analysis.

**Results:**

Overweight and obese adults with and without metabolic syndrome had higher fetuin-A levels were than the controls. The results of this study indicated a positive correlation between fetuin-A and lipid profile, anthropometric parameters, 12 h fasting blood glucose and blood pressure. Contrarily, HDL-C exhibited a negative correlation with fetuin-A.

**Conclusions:**

Fetuin-A is the first hepatokine associated with metabolic diseases. It controls the entire energy homeostasis of the body probably by regulating glucose and lipid metabolism. The current data belief is that fetuin-A may be a promising biomarker for predicting metabolic syndrome and its associated disorders particularly in overweight and obese adults.

## Introduction

Obesity is an issue of growing concern in many low- and middle-income countries. There is compelling evidence that in every region the number of people living with obesity is higher than that of people who are underweight. Emerging evidence from the World Obesity Federation report suggested that globally, one billion people including one in seven men and one in five women will be living with obesity by 2030 ^(1)^. Reports from different parts of India indicated that the prevalence rates of obesity/ overweight among the women and men are 36.2% and 38.4% respectively ^(2)^. Considerable evidence suggests that obesity is a commonly neglected public health problem and is always associated with unemployment, social disadvantages and reduced socioeconomic productivity ^(3)^. A handful of information suggest that obesity particularly central obesity is the main risk factor for metabolic syndrome and metabolic syndrome is well established two fold increased risk factor for cardiovascular mortality, diabetes mellitus, and various cancer types such as endometrial, breast, ovarian, prostate, liver, gall bladder, kidney and colon cancers ^(4), (5), (6)^. Notably, the prevalence of metabolic syndrome varies worldwide, and it often correlates with the prevalence of obesity. In India the prevalence of metabolic syndrome has dramatically increased, it has been reported that the pooled overall prevalence of metabolic syndrome in the adult population is 30% ^(7)^. Accumulating evidence indicates that hepatokines have gained considerable interest. Hepatocyte secretes proteins called hepatokines such as fetuin-A which might influence the metabolic processes through autocrine, paracrine and endocrine signaling. Hepatokine dysregulation may lead to progression toward the metabolic syndrome, type 2 diabetes, inflammation, hypertension, and other diseases. Fetuin-A has been suggested to abate lipogenesis, and the underlying mechanism could be that it increases lipolysis particularly in adipocytes, thereby inducing obesity and insulin resistance ^(8)^. Fetuin-A is predominantly produced in the liver in adults and it the chief hepatokine to be associated with metabolic diseases ^(9)^. Human fetuin-A was previously known as α2-Heremans-Schmid glycoprotein, a 64-kDa glycoprotein found in serum at relatively high concentrations ^(10)^. It is well established that fetuin-A is predominantly secreted and expressed in the liver and adipose tissue ^(11)^. It is noteworthy that the liver may control the energy homeostasis of the whole body by influencing glucose and lipid metabolism through fetuin-A production ^(12), (13)^. The available medical literature emphasized that circulating fetuin-A levels are increased in overweight/obesity and in obesity related disorders, such as metabolic syndrome, diabetes, and cardiovascular diseases ^(14), (15), (16), (17)^. However some studies did not find a good association between fetuin-A and body mass index (BMI). Fetuin-A stimulates the synthesis of proinflammatory cytokines in the adipocytes and macrophages ^(18)^ and might act as a ligand for Toll-like receptor 4, including insulin resistance by enabling free fatty acids to activate Toll-like receptor 4 signaling ^(19)^. It is also considered as a novel marker to link between obesity and its complications such as metabolic syndrome ^(8)^. Nevertheless, the biological mechanism of fetuin-A in insulin resistance remains unclear. Therefore, we aimed to investigate the role of serum fetuin-A in overweight and obese adults with and without metabolic syndrome in the northeastern Indian population. We also explored the correlation of serum fetuin-A with biochemical and anthropometric parameters. To the best of our knowledge, there are no studies, examining the role of fetuin-A levels in overweight and obese adults with and without metabolic syndrome particularly, those of the northeastern Indian population. It is better to identify adults with overweight and obesity and their prospect of metabolic syndrome at the initial stage to avert the eventual development of metabolic syndrome and its associated disorders.

## Materials and Methods

The study group consisted of 200 subjects aged 20-70 years (50 subjects with overweight, 50 subjects with obesity without metabolic syndrome, 50 subjects with obesity with metabolic syndrome and 50 healthy controls) from the Government Medical College & Hospital (Tomo Riba Institute of Health & Medical Sciences), Naharlagun, Papum Pare District, Arunachal Pradesh. A detailed questionnaire recorded the medical history of all the participants. Study approval was obtained from the Institutional Ethics Committee before the commencement of the study. Furthermore, written informed consent was obtained from all the participants. Adults with secondary causes of obesity; adults with known history of diabetes, cardiovascular diseases, cancer, and relevant drug treatments; and adults receiving treatment for any other comorbid conditions were excluded. Routine anthropometric measurements, such as height, weight, BMI, waist-to-hip ratio (WHR) and neck circumference (NC), were recorded. Weight was measured for all the subjects to the nearest 0.1kg using a beam balance.

Height was calculated to the nearest centimeter using a tape stuck to the wall. Waist circumference was measured at the level of the umbilicus, with the subject in the relaxed state and standing position. Hip circumference was measured at the widest point of the hips at the level of the greater trochanter with the subject in the standing position with both feet placed together. From the above measurements the WHR was calculated. NC was estimated using nonstretchable plastic tape in the midway of the neck between the midcervical spine and midanterior neck to within 1mm with the subject standing upright with a laryngeal prominence, and the dimension was recorded below the prominence. Subjects were considered with BMI between 25 and 29.9 were considered as overweight and those with BMI above 30 as obese. Subjects with metabolic syndrome were identified using the IDF criteria. These criteria define metabolic syndrome as having central obesity (waist circumference >90 cm in men, and >80 cm in women) along with two of the following factors:- 1) TG 150 mg/dL or higher 2) HDL levels < 40mg/dL in men or < 50 mg/dL in women 3) BP 130/85 mmHg or higher, and 4) fasting blood glucose > 100 mg/dL. Blood pressure levels (both systolic and diastolic) were documented for the study subjects using a mercury sphygmomanometer.

Blood samples were collected from all the subjects after a 12-h fasting. The serum was parted and all the samples were preserved at -20^0^C until analysis. The lipid profile included total cholesterol, low-density lipoprotein cholesterol, high-density lipoprotein cholesterol which were investigated using an enzymatic method and a fully automated analyzer (Agappe). Serum triglyceride and fasting blood glucose levels were analyzed using GPO-PAP and GOD-POD methods, respectively, in a fully automated analyzer (Agappe). All the samples for lipid profile and fasting blood glucose were immediately analyzed. Serum fetuin-A levels were measured using ELISA (Fine Test).

### Statistical analysis

The SPSS version 23.0; SPSS Inc; Chicago IL, USA was used to interpret the statistical data. For the descriptive research data, categorical variables were expressed as frequency and percentages, whereas continuous variables were expressed as mean ± standard deviation. *T*-test was employed for the numerical investigational data, chi-squared test for categorical data, and Pearson’s correlation for the correlation analysis 5% level of significance was applied.

## Results

The present study examined 200 subjects (50 healthy controls, 50 overweight, 50 obese without metabolic syndrome and 50 obese with metabolic syndrome). Of them 69 were men and 131 were women there average age was 20-70 years. A total of 48 subjects were excluded due to unwillingness to participate in the study, incomplete data or recent diagnosis any other disorder. The biochemical and other findings of the study subjects are presented in Table 1. The anthropometric parameters were found to be substantially increased in overweight adults and obese adults with and without metabolic syndrome. WHR was found to be nonsignificant in the overweight group. The serum fetuin-A levels were significantly higher in overweight (5.5 ± 1.2 ng/mL), obese without metabolic syndrome (10.7 ± 2.1 ng/mL) and obese with metabolic syndrome (32.9 ± 10.5 ng/mL) groups than in the controls (1.9 ± 0.8 ng/mL) (*P*<0.001). The serum fetuin-A levels were significantly positively correlated with BMI, WHR, NC, glucose, Total cholesterol, Triglycerides, Low-density lipoprotein cholesterol, Very low-density lipoprotein, Systolic blood pressure and Diastolic blood pressure. Furthermore, the serum fetuin-A levels were negatively correlated with the HDL-C levels. The associations between serum fetuin-A and other parameters for the enrolled participants are presented in Table 2. Men had slightly higher serum fetuin-A levels than women. The biochemical and anthropometric differences among men and women are presented in Table 3 and the affinity between serum fetuin-A and all the other parameters for men and women are displayed in Table 4. The socioeconomic status and physical activity of the participants were found to be almost identical. Of the 200 subjects only 6 were vegetarian (three men in the control, one woman in obese without MS, two men in obese with MS).

**Table 1.** Comparison between the Control, Overweight and Obese with and without Metabolic Syndrome Groups.

Parameter	Control (N= 50)	Overweight (N= 50)	Obese without metabolic syndrome (N= 50)	Obese with metabolic syndrome (N= 50)
Age (Years)	41.5 ± 13.4	37.1 ± 10.9	40.6 ± 12.7	42.0 ± 9.2
BMI (Kg/m^2^)	21.5 ± 1.8	27.2 ± 1.2^**^	31.4 ± 1.9^**^	32.8 ± 2.7^**^
Waist circumference (cm)	76.2 ± 6.1	80.7 ± 6.3^†^	90.0 ± 9.1^**^	98.9 ± 7.7^**^
WHR	0.8 ± 0.1	0.8 ± 0.1^ †^	0.9 ± 0.0^**^	0.9 ± 0.1^**^
NC	14.1 ± 0.8	14.6 ± 0.7^*^	15.8 ± 1.0^**^	16.2 ± 1.1^**^
Glucose (mg/dL)	90.2 ± 8.8	95.2 ± 10.7^*^	94.9 ± 8.5^*^	108.5 ± 10.4^**^
TC (mg/dL)	167.5 ± 32.3	177.1 ± 37.7^ †^	190.4 ± 26.8^*^	219.6 ± 42.3^**^
TG (mg/dL)	121.9 ± 33.0	162.8 ± 6.8^**^	150.2 ± 41.2^**^	242.0 ± 9.61^**^
HDL-C (mg/dL)	48.8 ± 11.6	43.2 ± 9.1^*^	49.1 ± 6.2^ †^	40.6 ± 7.0^*^
LDL-C (mg/dL)	92.2 ± 26.2	104.3 ± 36.1^ †^	107.5 ± 22.4^*^	135.0 ± 40.5^**^
VLDL (mg/dL)	24.1 ± 6.8	32.5 ± 13.6^*^	29.8 ± 8.2^*^	48 ± 18.8^**^
S.B.P (mmHg)	116.2 ± 6.4	117.6 ± 5.6^ †^	117.8 ± 6.8^ †^	133.8 ± 10.7^**^
D.B.P (mmHg)	79.6 ± 6.7	79.4 ± 5.5^ †^	79.8 ± 6.8^ †^	87.8 ± 8.2^**^
Fetuin-A (ng/mL)	1.9 ± 0.8	5.5 ± 1.2 ^**^	10.7 ± 2.1 ^**^	32.9 ± 10.5 ^**^

**P<0.001, *P<0.005, † *P*>0.05 BMI-body mass index, WHR-waist-to-hip ratio, NC-neck circumference TC-total cholesterol, TG-triglyceride, HDL-C-high-density lipoprotein cholesterol, LDL-C-low-density lipoproteincholesterol, VLDL-very-low-density lipoprotein, S.B.P-systolic blood pressure, D.B.P-diastolic blood pressure.

**Table 2.** Pearson’s Correlation Analysis between Serum Fetuin-A and the Anthropometric, and Biochemical Variables of the Study Subjects.

Parameter	r value	p value
BMI	0.67	0.001
WHR	0.45	0.001
NC	0.50	0.001
Glucose	0.54	0.001
TC	0.46	0.001
TG	0.50	0.001
HDL-C	^_^0.22	0.002
LDL-C	0.40	0.001
VLDL	0.50	0.001
S.B.P	0.61	0.001
D.B.P	0.37	0.001

BMI-body mass index, WHR-waist-to-hip ratio, NC-neck circumference TC-total cholesterol, TG-triglyceride, HDL-C-high-density lipoprotein cholesterol, LDL-C-low-density lipoprotein cholesterol, VLDL-very-low-density lipoprotein, S.B.P-systolic blood pressure, D.B.P-diastolic blood pressure.

**Table 3.** Comparison between the Male and Female Participants from the Different Groups.

Parameter	Group I		Group II		Group III		Group IV	
	Males (N= 17)	Females (N= 33)	Males (N= 13)	Females (N= 37)	Males (N= 16)	Females (N= 34)	Males (N= 23)	Females (N= 27)
Age (Years)	42.1 ± 12.7	41.1 ± 13.9	37.6 ± 12.2	36.8 ± 10.6	42.1 ± 10.8	39.8 ± 13.5	41.5 ± 9.1	42.3 ± 9.5
BMI (Kg/m^2^)	21.7 ± 1.7	21.3 ± 1.7	27.0 ± 1.2^**^	27.2 ± 1.1^**^	30.8 ± 1.2^**^	31.7 ± 2.1^**^	33.1 ± 3.3^**^	32.5 ± 1.9^**^
WHR	0.78 ± 0.06	0.78 ± 0.06	0.8 ± 0.07^†^	0.81 ± 0.06^†^	0.86 ± 0.05^**^	0.86 ± 0.04^**^	0.88 ± 0.05^**^	0.87 ± 0.04^**^
NC	14.6 ± 0.69	13.8 ± 0.74	14.9 ± 0.65^†^	14.5 ± 0.68^*^	16.4 ± 1.0^**^	15.4 ± 0.87^**^	16.4 ± 1.0^**^	15.9 ± 1.0^**^
Glucose (mg/dL)	88.8 ± 8.0	90.8 ± 9.2	95.7 ± 15.1^†^	95.0 ± 8.8^†^	96.6 ± 11.6^†^	93.9 ± 6.5^†^	108.2 ± 9.8^**^	108.7 ± 11.0^**^
TC (mg/dL)	164.6 ± 37.5	168.9 ± 29.7	166.9 ± 40.4^†^	180.6 ± 36.5^†^	183.1 ± 25.8^†^	193.8 ± 26.9^**^	223.0 ± 41.0^**^	216.6 ± 43.9^**^
TG (mg/dL)	120.6 ± 34.3	122.5 ± 32.7	171.7 ± 61.9^*^	159.6 ± 70.6^*^	137.8 ± 18.2^*^	156.0 ± 47.5^**^	261.1 ± 132.1^**^	225.6 ± 45.1^**^
HDL-C (mg/dL)	48.5 ± 11.6	48.9 ± 11.7	41.3 ± 6.5^†^	43.8 ± 9.8^†^	47.0 ± 3.4^†^	50.1 ± 6.9^†^	39.3 ± 7.1^**^	41.7 ± 6.7^**^
LDL-C (mg/dL)	87.4 ± 29.7	94.5 ± 24.2	93.6 ± 33.6^†^	107.9 ± 36.6^†^	103.5 ± 25.5^†^	109.3 ± 20.9^*^	139.9 ± 38.1^**^	130.7 ± 42.5^**^
VLDL (mg/dL)	24.0 ± 6.9	24.1 ± 6.8	34.2 ± 12.3^*^	31.8 ± 14.1^*^	27.3 ± 3.6^†^	30.9 ± 9.4^*^	51.5 ± 25.8^**^	45.0 ± 9.0^**^
SBP (mmHg)	118.2 ± 8.0	115.1 ± 5.0	116.9 ± 6.3^†^	117.8 ± 5.3^†^	119.3 ± 6.8^†^	117.0 ± 6.7^†^	135.2 ± 11.6^**^	132.5 ± 9.8^**^
DBP (mmHg)	78.2 ± 6.3	80.3 ± 6.8	79.2 ± 4.9^†^	79.4 ± 5.7^†^	80.0 ± 6.3^†^	79.7 ± 7.1^†^	88.2 ± 10.2^**^	87.4 ± 5.9^**^
Fetuin-A (ng/mL)	1.91 ± 0.78	1.89 ± 0.76	5.7 ± 1.6^**^	5.4 ± 1.3^**^	10.7 ± 2.5^**^	10.6 ± 1.8^**^	34.1 ± 10.2^**^	31.8 ± 10.7^**^

**P<0.001, *P<0.005, †*P*>0.05 BMI-body mass index, WHR-waist-to-hip ratio, NC-neck circumference TC-total cholesterol, TG-triglyceride, HDL-C-high-density lipoprotein cholesterol, LDL-C-low-density lipoproteincholesterol, VLDL-very-low-density lipoprotein, S.B.P-systolic blood pressure, D.B.P-diastolic blood pressure.

**Table 4.** Pearson’s Correlation Analysis between Serum Fetuin-A and the Anthropometric, and Biochemical Variables for Males and Females.

Parameter	Males (N=69)		Females (N=131)	
	r value	p value	r value	p value
BMI	0.76	0.001	0.61	0.001
WHR	0.52	0.001	0.39	0.001
NC	0.47	0.001	0.49	0.001
Glucose	0.53	0.001	0.54	0.001
TC	0.51	0.001	0.43	0.001
TG	0.50	0.001	0.49	0.001
HDL-C	^_^0.31	^_^0.009	^_^0.16	^_^0.06
LDL-C	0.49	0.001	0.33	0.001
VLDL	0.50	0.001	0.50	0.001
S.B.P	0.57	0.001	0.62	0.001
D.B.P	0.40	0.001	0.35	0.001

BMI-body mass index, WHR-waist-to-hip ratio, NC-neck circumference TC-total cholesterol, TG-triglyceride, HDL-C-high-density lipoprotein cholesterol, LDL-C-low-density lipoproteincholesterol, VLDL-very-low-density lipoprotein, S.B.P-systolic blood pressure, D.B.P-diastolic blood pressure.

## Discussion

The prevalence of metabolic syndrome is rapidly increasing in India, particularly in urban areas, with the rates ranging from 11% to 41% in different parts of the country ^(20)^. The present study aimed to evaluate the risk of metabolic syndrome and its associated diseases in overweight and obese adults of the northeastern Indian population and to exemplify the association between fetuin-A and other routine biochemical and anthropometric parameters in overweight adults and obese adults with and without metabolic syndrome.

The results of this study showed increased levels of serum fetuin-A in overweight adults and obese adults with and without metabolic syndrome. The available information strongly suggests that fetuin-A is involved in the decrease the lipogenesis and increase in lipolysis in the adipose tissue thereby stimulating obesity and insulin resistance. We hypothesized that liver might influence body energy homeostasis by governing the glucose and lipid metabolism and our data are consistent with those reported by other studies ^(8), (12)^. It is generally believed that elevated fetuin-A levels in obesity with metabolic syndrome is a significant predictive factor for type 2 diabetes and myocardial infarction/stroke ^(14), (15), (16), (17)^. Basic anthropometry parameters such as BMI and NC were markedly elevated in overweight and obese adults with and without metabolic syndrome. Contrarily, the WHR was nonsignificant in overweight adults and elevated in obese adults with and without metabolic syndrome. Fetuin-A levels were positively correlated with anthropometric parameters. Accordingly, it is possible that higher fetuin-A levels are involved in the pathogenesis of obesity and obesity related disorders including inflammation and metabolic syndrome ^(21)^. However, one study did not find an association between fetuin-A and BMI ^(22)^. Our findings suggest that fetuin-A could be a useful novel marker in clinical application in the near future for the evaluation of metabolic syndrome. The results of our study also showed elevated fasting blood glucose levels in overweight adults and obese adults with and without metabolic syndrome. A strong association between fetuin-A and fasting blood glucose was also observed. Our observations raise the possibility that fetuin-A is involved in both obesity and type 2 diabetes. It is also possible that prospective changes in fasting blood glucose are associated with fetuin-A and involved in the pathophysiology of type 2 diabetes and that changes in fetuin-A level precede the onset of type 2 diabetes ^(23), (24)^. Our findings indicate that increases in the concentrations of fasting blood glucose and free fatty acids in the blood of an individual with Metabolic syndrome may trigger the binding of NF-κB to the promoter of fetuin-A, and then stimulate the expression of circulating fetuin-A mRNA to further promote the synthesis and secretion of fetuin-A ^(25)^. High fetuin-A levels predict the occurrence of diabetes mellitus particularly type 2 diabetes, independently of any other markers of insulin resistance ^(26), (27)^. However, conflicting reports have been published based on the role of fetuin-A and its complications of diabetes. Some studies reported lower fetuin-A levels in individuals with type 2 diabetes mellitus ^(28)^. In this study, we observed unfavorable lipid profile and higher odds of dyslipidemia. Serum fetuin-A exhibited positive correlations with TC, TG, LDL-C and VLDL. Our results indicated an inverse association between fetuin-A level and HDL-C. Numerous recent cross sectional studies have demonstrated the desirable pathogenetic role for serum fetuin-A in the confederation with the insulin resistance possible mechanism could be by inhibiting insulin receptor leads to elevated lipolysis and discharge of free fatty acids from adipose tissue. This may in turn lead to increased production of VLDL, hypertriglyceridemia, which may enhance HDL clearance from the circulation, thereby potentially leading to the atherogenic lipid profile and it has been associated with cardiometabolic risk factors ^(15), (29), (30), (31)^. However, some studies did not find a good association between fetuin-A and lipid profile ^(22)^. Although the SBP and DBP values were nonsignificant in the overweight and obese without metabolic syndrome groups. SBP and DBP were markedly elevated in the obese with metabolic syndrome group. Furthermore, the serum fetuin A levels were positively correlated with SBP and DBP. Our observations imply that fetuin-A and metabolic syndrome have a functionally relevant association with obesity and play a pivotal role in the pathophysiology of metabolic disease ^(32), (33)^. The potential limitation of our study was the lack of assessment of the physical activity and dietary habits of the study participants.

The hypothesis of this study is that elevated fetuin-A levels in overweight and obesity with and without metabolic syndrome can influence various metabolic processes and play a pivotal role in inflammation. Obesity particularly central obesity is the prominent risk factor for insulin resistance, metabolic syndrome, type 2 diabetes mellitus, cardiovascular diseases and cancer. Diagnosis at an early stage is important to employ lifestyle and risk factor modification. We believe that serum fetuin-A is a useful biomarker for the early detection of metabolic syndrome and its associated diseases in overweight and obesity. Future research involving a larger sample size should elucidate the exact mechanism of fetuin -A in overweight and obese adult population.

## Article Information

### Conflicts of Interest

None

### Acknowledgement

We thank all the subjects for their co-operation and encouragement in carrying out the study. Heartfelt thanks to the Technical staff of the Department of Biochemistry and the Immunology, Dr.Iyir Gadi Tutor in Biochemistry at TRIHMS, Naharlagun. Heartfelt thanks to the Dr. Libe Nyorak Medicine Specialist, TRIHMS. We wish to thank Mr. Janaki Raman, Statistician, and Dr.Moji Jini, Director, Dr.Shyamal Kumar Bhattacharya Dean TRIHMS Naharlagun, India for all their support and co-operation in the conduction of this study.

### Author Contributions

Author 1:- Sruti Eswar, Working as an Assistant Professor in the Department of Biochemistry, TRIHMS, and registered for Part-Time Ph.D in Biochemistry (Faculty of Medicine) at Sri Balaji Vidyapeeth (Deemed-to be-University). The current research work was carried out by her under the supervision of the other co-authors.

Author 2:- Dr.Balaji Rajagopalan, Professor & HOD of Biochemistry, Sri Balaji Vidyapeeth (Deemed- to be- University), Ph.D Guide of the candidate (Sruti.E). He was involved in the conception, design, analysis, interpretation of data and drafting the article.

Author 3:- Dr.Kenyi Ete, Associate Professor, TRIHMS, Co-Guide of the candidate. He was involved in the acquisition of data, analysis, interpretation of data and drafting the article.

Author 4:- Dr.Srinivasa Nageswara Rao Gattem, Professor & HOD of Biochemistry, TRIHMS, Research Advisory Member from the department who was also actively involved in the acquisition of data, analysis, interpretation of data and drafting the article.

### Approval by Institutional Review Board (IRB)

IRB code: IEC No.TRIHMS/ETHICS/01/2019-20/14, Institution: Tomo Riba Institute of Health & Medical Sciences.
